# A variational algorithm to detect the clonal copy number substructure of tumors from scRNA-seq data

**DOI:** 10.1038/s41467-023-36790-9

**Published:** 2023-02-25

**Authors:** Antonio De Falco, Francesca Caruso, Xiao-Dong Su, Antonio Iavarone, Michele Ceccarelli

**Affiliations:** 1 Department of Electrical Engineering and Information Technology (DIETI), University of Naples ‘Federico II’, 80128 Naples, Italy; 2 BIOGEM Institute of Molecular Biology and Genetics, 83031 Ariano Irpino, Italy; 3 Biomedical Pioneering Innovation Center (BIOPIC), School of Life Sciences, Peking University, 5 Yiheyuan Road, Haidian District, 100871 Beijing, China; 4 Sylvester Comprehensive Cancer Center, University of Miami, Miller School of Medicine, Miami, FL USA; 5 Department of Neurological Surgery, University of Miami, Miller School of Medicine, Miami, FL USA

**Keywords:** Computational biology and bioinformatics, Mathematics and computing, Cancer genomics, Cancer microenvironment, Tumour heterogeneity

## Abstract

Single-cell RNA sequencing is the reference technology to characterize the composition of the tumor microenvironment and to study tumor heterogeneity at high resolution. Here we report Single CEll Variational ANeuploidy analysis (SCEVAN), a fast variational algorithm for the deconvolution of the clonal substructure of tumors from single-cell RNA-seq data. It uses a multichannel segmentation algorithm exploiting the assumption that all the cells in a given copy number clone share the same breakpoints. Thus, the smoothed expression profile of every individual cell constitutes part of the evidence of the copy number profile in each subclone. SCEVAN can automatically and accurately discriminate between malignant and non-malignant cells, resulting in a practical framework to analyze tumors and their microenvironment. We apply SCEVAN to datasets encompassing 106 samples and 93,322 cells from different tumor types and technologies. We demonstrate its application to characterize the intratumor heterogeneity and geographic evolution of malignant brain tumors.

## Abstract (web summary)

The inference of clonal architectures in cancer using single-cell RNA-seq data remains challenging. Here, the authors develop SCEVAN, a variational algorithm for copy number-based clonal structure inference in single-cell RNA-seq data that can characterise evolution and heterogeneity in the tumour and the microenvironment.

## Introduction

Understanding intratumor heterogeneity and the interactions between tumor cells and the immune system is the critical step to explaining treatment failure and plays a crucial role in studying tumor growth and evolution^1,2^. Single-cell RNA sequencing (scRNA-Seq) has been successfully used to identify multiple transcriptional programs activated in a single tumor^3–5^ and to prioritize key regulators of tumor-host interaction^6^. To study the complexity of lineage identity, differentiation, and proliferation of tumor cells and the impact of stromal and immune components, a large number of unsorted cells from tumor biopsies are subject to whole transcriptomics profiling and then classified as malignant cells, stromal cells, and immune cells, and further stratified into different compartments according to either expression of specific markers^6^, and the orchestrated activation of pathways^5^. The distinction of malignant from non-malignant cells is a critical step in the follow-up analysis of scRNA-seq tumor datasets. The basic idea to solve such a problem relies on estimating common copy number alterations that characterize transformed cells. The copy number profiles are obtained by considering the gene expression profiles of each cell as a function of the genomic coordinates. The moving average smoothing of the gene expression function is then clustered in malignant and non-malignant cells. One of the most successful methods based on this approach is the inferCNV algorithm^4^. One drawback is that the clusters of reference cells require manual identification, usually with a combination of approaches^7,8^. Moreover, inferCNV and similar methods^4,9^ are particularly suited for smart-seq data having high coverage and relatively low throughput, whereas they exhibit sub-optimal performances on droplet-based methods with very sparse coverage depth and higher throughput^10^. An approach to overcome these limitations is represented by the CopyKAT method^11^ that automatically classifies malignant and non-malignant cells. It was successfully applied to analyze the clonal substructure of three triple-negative breast tumors. However, the classification produced by CopyKAT can be affected by a wrong identification of normal cells and, similarly to other methods, was not designed to perform a complete automatic identification of the clones, reporting their breakpoints, the specific and shared alteration, and a clonal deconvolution in a complete end-to-end pipeline.

Here, we present Single CEll Variational Aneuploidy aNalysis (SCEVAN), a variational algorithm for automatically detecting the clonal copy number substructure of tumors from single-cell data. Our method automatically segregates malignant cells from non-malignant cells, and clusters of malignant cells are then analyzed through an optimization-based joint segmentation algorithm. We exploit the notion that all the cells in a given copy number clone share the same breakpoints with the smoothed expression profile of every individual cell providing support for the definition of the copy number profile of each subclone. Therefore, joint segmentation allows the enhancement of systematic biases leading to the emergence of consistent breakpoints. Afterward, SCEVAN performs a complete downstream analysis to automatically identify tumor subclones, classifying their specific and shared alterations up to a clone phylogeny. The joint segmentation algorithm implemented in SCEVAN is based on a variational framework developed in the field of Computer Vision, making use of the Mumford–Shah energy model^12^ that has already been successfully applied to detect copy number alterations in matched tumor–normal pairs of high-density comparative genomic hybridization arrays^13^ and used to detect fusion breakpoints^14^. Moreover, its joint version was developed to identify recurrent copy number alterations in large tumor cohorts^15,16^. Here, we benchmark the output of SCEVAN against state-of-the-art methods and show that SCEVAN exhibits faster and more accurate performance on synthetic and real data with reference copy number from bulk tumor profiling. Finally, we used SCEVAN to characterize the clonal substructure in multiple scRNA-seq glioma and head and neck cancer datasets.

## Results

### SCEVAN workflow

**Fig. 1:**
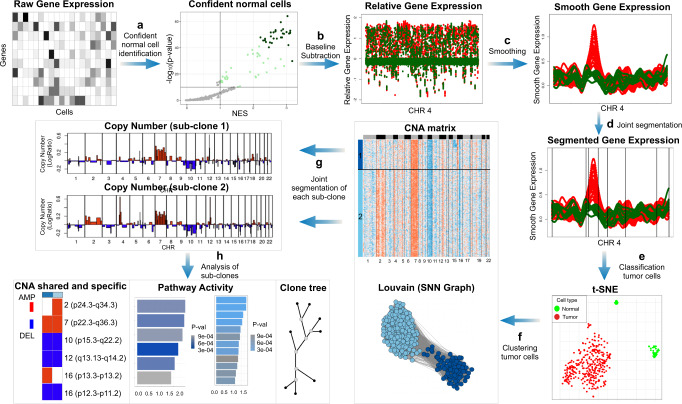
SCEVAN Workflow. SCEVAN starts from the raw count matrix removing irrelevant genes and cells. **a** Identification of a small set of highly confident normal cells. **b** Relative gene expression obtained from removal of the baseline inferred from confident normal cells. **c** Edge-preserving nonlinear diffusion filtering of relative gene expression. **d** Segmentation with a variational region-growing algorithm. **e** Identification of normal cells as those in the cluster containing the majority of confident normal cells. **f** Identification of possible subclones using Louvain clustering applied to a shared nearest-neighbor graph of the tumor cells. **g** Segmentation with a variational region-growing algorithm applied to each subclone. Segments are then classified in five copy number states. **h** Analysis of subclones including clone tree, pathway activities (GSEA was performed for each subclone using fgseaMultilevel which calculates *P* values based on an adaptive multilevel splitting Monte Carlo scheme), and characterization of shared and specific alterations.

The workflow of SCEVAN (Fig. 1) starts from the raw count matrix with genes on rows and cells on columns. The input count matrix is log-transformed and then pre-processed by removing cells with a low number of detected transcripts and selecting the most expressed genes. A set of highly confident non-malignant cells are identified and used to determine a copy number baseline and to compute the relative matrix removing the baseline (Steps A and B). This matrix undergoes an edge-preserving nonlinear diffusion filter assuming a piecewise smooth function as the underlying model (Step C). The smoothed matrix is then segmented using the joint segmentation algorithm to obtain a copy number matrix (Step D). SCEVAN discriminates the normal cells from tumor cells as those falling in the cluster containing the highest number of confident normal cells (Step E). The different subclones are obtained by analyzing the clusters of the tumor cells in the Copy Number Matrix as detailed in the Methods (Step F). Then each cluster is segmented independently from the smoothed matrix to obtain a copy number profile for any subclone (Step G). The segments are classified in one of five predefined copy number states: deletion, loss, neutral, gain, or amplification, using a majority vote applied to a mixture model classification of each cell. Finally, SCEVAN characterizes truncal, shared, and clone-specific alterations comparing different clusters, performing enrichment analysis up to a clone phylogeny (Step H).

### Malignant cell classification on synthetic data

To quantitatively evaluate the accuracy of SCEVAN in discriminating malignant from non-malignant cells, we generated 500 synthetic matrices with known tumor/normal classification (Supplementary Data 3). We used a Multiple Myeloma dataset containing 17,267 malignant plasma cells and 57,719 immune cells of Liu et al.^17^. Based on the specific markers used by the authors, we classified cell clusters in eight immune compartments and tumor cells of each patient. We trained a scDesign2^18^ model for each cell type, specifically eight immune and 14 malignant models, one for each sample. The synthetic scRNA-seq matrices were randomly generated by choosing the following parameters: the number of total cells (between 300 and 1000), the tumor purity (between 5 and 100%), the number of cells for each immune cell type, and the scDesign2^18^ malignant model from one of the 14 samples. The generated matrices had on average 94% of zero values. We further added dropout noise at different levels to each simulated sparse count matrix. Dropout simulations have probabilities conditioned on mean gene expression, such that lowly expressed genes have a higher likelihood of dropout than highly expressed genes. This type of noise is added using SPLATTER^19^ which uses a logistic function to produce a probability that a count should be zero. The logistic function is defined by a midpoint parameter, *x*_0_, the logarithm of the expression level at which 50% of cells are replaced with zero. The probability of a zero for each gene is then used to randomly replace some of the simulated counts with zeros using a Bernoulli distribution. We used three noise levels corresponding to the values of *x*_0_ = −2, −1, 0, that respectively replace 7%, 17%, and 31% of non null values with a 0. We applied SCEVAN and CopyKAT to these synthetic matrices containing a total of 322,687 cells (Supplementary Fig. 1), obtaining with SCEVAN a mean F1 score of 0.948, 0.943, 0.909, 0.824 and with CopyKAT 0.798, 0.792, 0.763, 0.726, for no noise and for each level of noise, respectively. It is worth noticing that in some cases both methods can obtain a very low F1 score, this is due to the fact that in cases of a an erroneous identification of the cluster of a normal cell, for example, a cluster of tumor cells is named as the reference normal, than a complete misclassification can happen and an F1 score close to zero is obtained.

### Malignant cell classification accuracy on real data

**Fig. 2:**
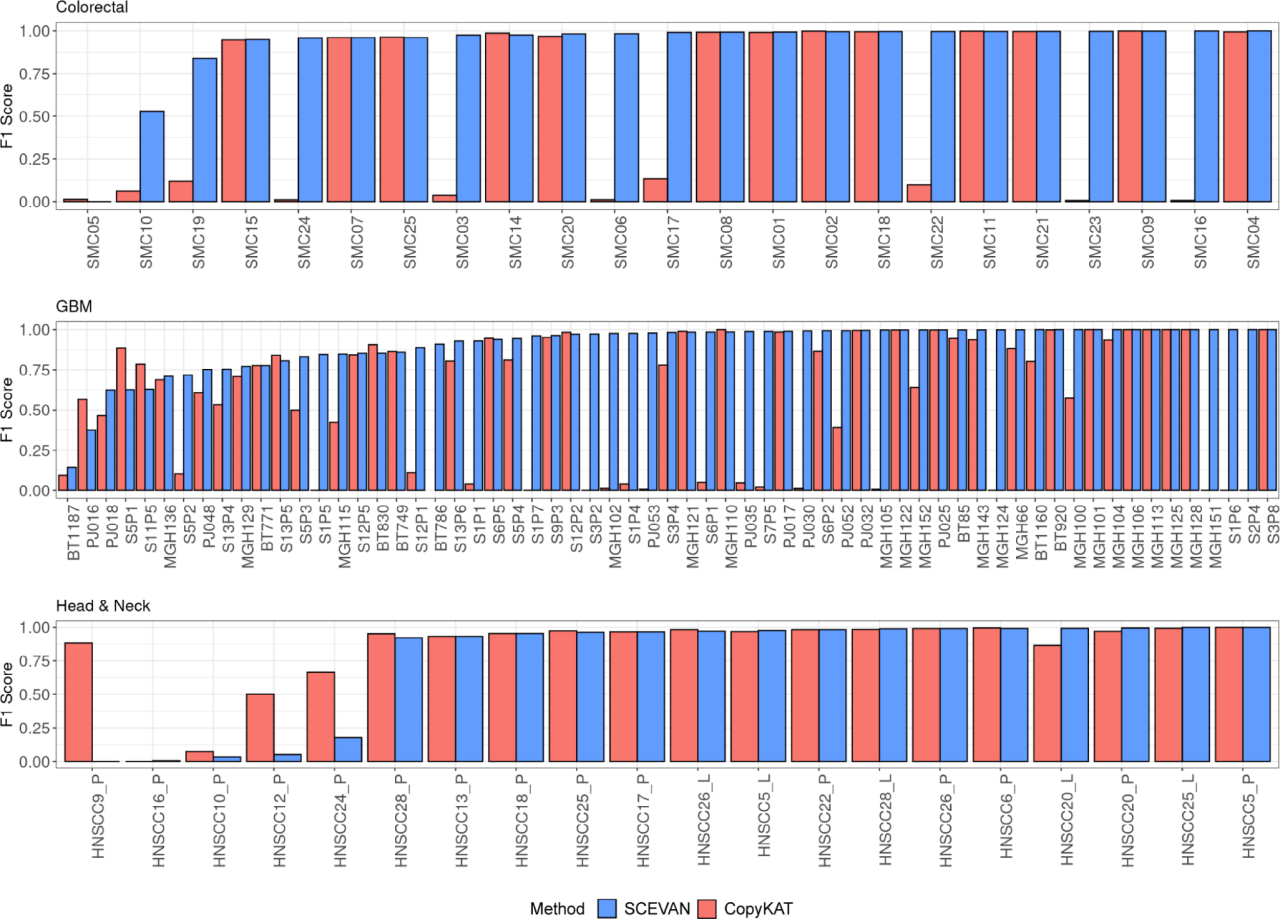
Benchmark of malignant cell classification task. F1 score obtained with SCEVAN and CopyKAT^11^ in the classification of malignant and non-malignant cells for each cancer type. Colorectal cancer^20^ *n* = 47,285 cells examined over 23 scRNA-seq independent experiments, Glioblastoma^7, 10, 22^ *n* = 40,320 cells examined over 63 scRNA-seq independent experiments, Head and Neck Squamous Cell Carcinomas^21^ *n* = 5717 cells examined over 20 scRNA-seq independent experiments (Supplementary Data 2). Source data are provided as a Source Data file.

We also evaluated the accuracy of non-malignant cell classification on real data, we applied our tool to several public datasets^7,10,20–22^ of three different cancer types of scRNA-seq data (Glioblastoma (GBM), Head and Neck Squamous Cell Carcinomas (HNSCC), Colorectal cancer) and from different sequencing technologies (Smart-seq2, 10X Chromium), classifying a total of 106 samples and 93,322 cells (Supplementary Data 2). In all the considered datasets, the identification of the non-malignant cell has been reported by the authors through manual curation based on a combination of approaches using copy number profile^4^, clustering, and cell markers. We compared our results in terms of F1 score^23^ with those obtained by using CopyKAT^11^. SCEVAN, as shown in Fig. 2, achieves a better classification score in 63% of the samples, whereas CopyKAT performs better than SCEVAN in 23% of the samples. The F1 score for all samples obtained with SCEVAN is 0.90 in contrast to the F1 score of 0.63 obtained with CopyKAT. SCEVAN shows a low F1 SCORE in samples with very few tumor cells (between 1 and 15), present mostly in the case of Head & Neck cancer dataset (Supplementary Data 2). For one of the samples (BT786), we could not get the results from CopyKAT due to crashes.

Collectively, these results confirm that SCEVAN can accurately discriminate between tumor and normal cells in different solid tumors using the copy number profiles inferred from scRNA-seq.

### Segmentation accuracy on synthetic data

To perform a quantitative evaluation of the segmentation results, we generated a synthetic dataset modeling two realistic scenarios: Scenario I, with just clonal alterations and all malignant cells share the same alterations; Scenario II, where there are some clonal alterations shared by all cells and also two populations of malignant cells having subclone-specific alterations. For both scenarios, we generated synthetic matrices with different levels of magnitude of the synthetic copy number alterations, starting from matrices previously obtained using scDesign2^18^. We considered only normal diploid cells and randomly alter genomic regions generating synthetic aneuploid cells.

For each matrix, we randomly choose the number of aneuploid cells (between 30 and 90% of total cells), the number of alterations (between 1 and 10), the central position of each alteration (between 1 and the number of total genes), the number of genes belonging to each alteration (between 50 and 1000), and in the case of scenario II the assignment of each cell to one of the two subclones.

To generate synthetic amplification (deletion), we increase (decrease) the count values of the genes belonging to the alteration. Specifically, we draw a uniform random value *ρ* in (0, *α*) and replace each gene count *x*_*ij*_ by *x*_*ij*_(1 + *ρ*) for amplifications and *x*_*ij*_/(1 + *ρ*) for deletions. Therefore, we increase/decrease the counts of the genes belonging to the alteration by a percentage between 0 and 100*α*%. We performed for each scenario four experiments corresponding to *α* = 2, 3, 4, generating for each scenario, and value of *α*, 100 matrices.

To define an appropriate evaluation metric for the segmentation produced by various segmentation algorithms, as previously suggested^24^, we scored as True Positive (TP) the breakpoints that lie within a tolerance threshold of distance (e.g., 20 genes) from the true breakpoints, and a false negative (FN) if there are no breakpoints in this tolerance area. The synthetic dataset was used to compare the accuracy of SCEVAN and CopyKAT, we also considered other segmentation approaches such as GFLars^24^ a method optimizing a squared loss and a regularization term based on group LASSO, and GenoCN,^25^ a method based on HMM segmentation. The details about the adopted parameters for this comparison are reported in the “Methods” section.

Using a threshold of 20 genes, SCEVAN obtains significantly higher F1 scores than other methods in each scenario and experiment (Supplementary Fig. 2). It is interesting to note that in some cases SCEVAN, as well as the other tools, gets a low score. This is due to a several factors. When all the breakpoints are identified at a distance greater that the tolerance threshold, or the method fails to identify most the alterations, then the corresponding classification score is close to zero. Moreover, since the synthetic matrices, as well the synthetic alterations, are randomly generated, it is possible that the alterations are located in regions where the average gene expression is low. In such cases, even for high amplitude of the alteration (the parameter *α*), the segmentation task becomes extremely challenging with the possibility to low detection accuracy.

The role of the parameters on the performance of the considered segmentation methods needs also to be investigated. In general, segmentation algorithms adopt some regularization parameters to control the amount of smoothing and the coarseness of the segmentation, such as the parameter *β* for SCEVAN that controls the convergence of the hierarchical region-merging procedure and defines a stopping criterion for the increasing sequence of the regularization parameters (“Methods”) and KS.cut for CopyKAT. Since an exhaustive exploration of the parameters for the considered algorithms may lead to over-optimistic results which are difficult to replicate in scenarios with real data, we use a dynamic programming approach that progressively selects optimal subsets of the breakpoints reported by a given method^24^ (jpruneByDP procedure of the jointseg Bioconductor package). With this setting, it is possible to compute a precision-recall (PR) curve for the output of various algorithms varying the size of selected optimal subsets of breakpoints. Here, we computed the mean area under the PR curve (AUC) as a function of the tolerance parameter for 100 simulated matrices at different levels of the magnitude of alteration *α* (Supplementary Fig. 3).

We observed that SCEVAN reaches consistently better AUC than the other segmentation methods and as the *α* parameter increases, i.e., when the steps in the genomics alterations are more noticeable, the improvement is even more evident.

We also evaluate the performance varying the segmentation parameters of SCEVAN and CopyKAT. For CopyKAT, we vary the parameter KS.cut in the interval suggested by the authors (0.05, 0.10, 0.15, 0.20, 0.25, 0.30, 0.35, 0.4), and for SCEVAN we vary the parameter *β* (0.1, 0.5, 1.0, 1.5, 2.0, 2.5, 3.0, 4.0). In both cases, the increase of these values results in coarser segmentations. PR curves are calculated for matrices with different *α* (2, 3, and 4), with clonal and subclonal scenarios, and using different tolerance values (10, 20, 30, and 40 genes). This analysis also confirms that SCEVAN’s accuracy is higher even with varying parameters and tolerance values (Supplementary Fig. 4). The results above refer to a limited number of alterations (between 1 and 10), we have observed that the overall accuracy is not significantly influenced by the number of simulated genomic alterations. Rather, it is influenced by the magnitude of the alteration *α* and the local distribution of the smoothed gene expression signal around the discontinuities induced by the breakpoints.

In the experiments reported in the sequel, we use the default value (*β* = 0.5) to produce slightly finer segmentations on real data accounting for more focal lesions. For the clonal analysis, the algorithm uses a slightly larger value (*β* = 3.0) to reduce the effect of the noise in the final output. Finally, the synthetic dataset is publicly available and could serve as a reference benchmark for other single-cell CNV inference algorithms.

### Segmentation accuracy using reference data

**Fig. 3:**
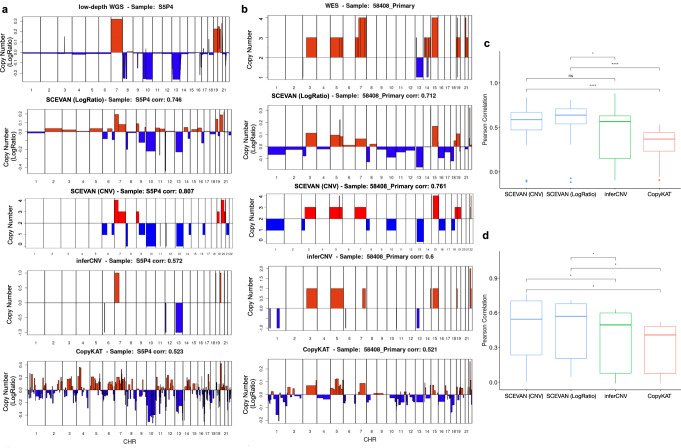
Benchmark of inferred copy number profile. **a**, **b** Copy number profile inferred with SCEVAN (segment mean (LogRatio) and CNV status), inferCNV, CopyKAT, the corresponding ground truth from low-depth WGS of sample S5P4^22^ and from WES of sample 58408 Primary^17^. **c**, **d** Boxplots show the median as center, the lower and upper hinges that correspond to the 25th and the 75th percentile, and whiskers that extend to the smallest and largest value no more than 1.5*IQR. Values that stray more than 1.5*IQR upwards or downwards from the whiskers are considered potential outliers and represented with dots. Significance was computed by a two-sided Wilcoxon signed-rank test (ns: *P* value > 0.05, *P value < = 0.05, ****P value <= 0.0001). **c** Pearson correlation between the copy number inferred with different methods and the ground truth from low-depth WGS for 26 samples^22^. SCEVAN obtains a significantly higher correlation than CopyKAT (LogRatio *P* value 1.3*e*−05 and CNV status *P* value 3.0*e*−07) and inferCNV (LogRatio *P* value 0.02). **d** Pearson correlation with the ground truth from WES for seven samples^17^. SCEVAN obtains a significantly higher correlation than CopyKAT (LogRatio and CNV status *P* value 0.016) and inferCNV (LogRatio *P* value 0.016 and CNV status *P* value 0.031). Source data are provided as a Source Data file.

After evaluating the accuracy of our method in the identification of the copy number breakpoints on synthetic data, we evaluated its accuracy on real datasets where we have both the single-cell RNA-seq and reference copy number profiles obtained from bulk DNA sequencing. Since, in this case, we are using real single-cell datasets, here we compare results produced by SCEVAN, inferCNV, and CopyKAT. Since CopyKAT returns just the segment mean, whereas the output of inferCNV is the inferred copy number status, when comparing both methods with SCEVAN we use both the segment mean, mentioned hereafter as LogRatio, and the copy number status called by the mixture model algorithm (“Methods”). We use as ground truth 26 samples of a Glioblastoma multiregional dataset^22^ with the CNV status from low-depth whole-genome sequencing (WGS) on the bulk biopsies (Fig. 3c) and seven samples (81012 Primary, 59114 Relapse-1, 58408 Primary, 58408 SMM, 27522 Primary, 57075 Relapse-1, and 37692 Primary) of Multiple Myeloma (MM) dataset^22^ with the CNV Status obtained using whole-exome sequencing (WES) on the bulk biopsies (Fig. 3d). We re-sampled the output of SCEVAN, CopyKAT, and inferCNV to the same resolution of the ground truth by taking one value every 1 Mb (“Methods”). The boxplots of Fig. 3 show the Pearson correlation between the inferred copy number profiles and the reference copy number obtained in all samples. SCEVAN as segment mean (LogRatio) has a mean correlation of 0.57 (max 0.81) on the multiregional GBM dataset and 0.44 (max 0.71) on the MM dataset. The copy number call of SCEVAN has a mean correlation of 0.54 (max 0.84) on the multiregional GBM dataset and 0.46 (max 0.76) on the MM dataset. CopyKAT has a mean correlation of −0.03 (max 0.52) on the multiregional GBM dataset and 0.29 (max 0.52) on the MM dataset. Whereas inferCNV has a mean correlation of 0.44 (max 0.88) on the multiregional GBM dataset and 0.35 (max 0.63) on the MM dataset.

Since inferCNV does allow automatic identification of the non-malignant cells, for the generation of these results, we used the set of non-malignant cells classified by SCEVAN. The lower accuracy of CopyKAT is probably due to the wrong classification of malignant and non-malignant cells. However, since the misclassification of normal cells could be eventually corrected by manual inspection, instead of using the whole multiregional dataset,^22^ we performed the same comparison using just the samples where CopyKAT achieves an F1 classification score above 0.50. This comparison evaluated the accuracy of segmentation on real-world data, limiting the effect of malignant/non-malignant misclassification. On the 13 samples where CopyKAT reaches the best classification results, we obtained a median correlation between the inferred CNV profile and the CNV from the bulk WGS of 0.648 and 0.309 for SCEVAN and CopyKAT respectively, as reported in Supplementary Fig. 5a.

As a further comparison, we run CopyKAT using the non-malignant cells identified by SCEVAN. With this approach, CopyKAT obtained a much higher correlation with the ground truth. On the 26 samples of the GBM multiregional dataset,^22^ it achieved a mean correlation of 0.33, as shown in Supplementary Fig. 5b. However, using the same classification of non-malignant cells, SCEVAN achieves a significantly higher correlation (*P* value 1.3*e*−5) than CopyKAT.

We also evaluated the robustness of the segmentation with respect to misclassification of the normal cells. We randomly removed from the reference control cells several cells at steps of 5%. We used eight samples from the GBM multiregional dataset^22^. As shown in Supplementary Fig. 6, SCEVAN is robust to a high percentage of misclassified cells. The correlation of the copy number variation profile of the malignant cells with the ground truth remains stable for errors less than 60% and, in some cases, up to 95%. These results further confirm the robustness of the segmentation method for the misclassification of normal cells.

These data indicate that SCEVAN accurately infers DNA copy number profiles from high-throughput scRNA-seq data.

### Computational efficiency comparison

SCEVAN is also particularly efficient since the main segmentation step is based on a greedy region-growing algorithm. To validate its performance in terms of computational efficiency, we compare the classification step of the malignant cells and the segmentation step separately. In the former case, the direct comparison of the execution times showed that SCEVAN is 2–7× faster (Supplementary Fig. 7a) in the discrimination phase between malignant and non-malignant cells. Afterward, when we compare the time required for segmentation, on the multiregional GBM dataset,^22^ SCEVAN is 2× faster than CopyKAT and 5× than inferCNV, instead for the Multiple Myeloma data^17^, sequenced with 10x Genomics technology, CopyKAT becomes particularly slow, due to large number of cells. Specifically, as shown in the Supplementary Fig. 7b, SCEVAN is 11× faster than inferCNV and 19× than CopyKAT.

These results show that the greedy segmentation algorithm implemented in SCEVAN is particularly efficient with respect to other tools for copy number inference from scRNA-seq.

### Intratumoral heterogeneity in glioblastoma

Glioblastoma (GBM) is the most aggressive form of brain tumor. It is characterized by high heterogeneity, with several clonal and subclonal tumor cell populations, glioma stem cells, and an immuno-repressive tumor microenvironment^7,26,27^.

SCEVAN can automatically infer clonal substructure from single-cell data by analyzing the clusters of the CNA matrix that show significantly different genomic alterations (“Methods”). As an application of this approach, we considered one of the samples reported in a recent study^7^, the MGH105 sample. SCEVAN identifies four sub-populations that have different alterations on chromosome 6 (Supplementary Fig. 8). Interestingly, whereas canonical scRNA-seq processing analyses could not reach the resolution for the identification of four subclones^7^, instead the existence of these subclones had been previously described through the application of DNA single-cell DNA methylation platforms^26^.

**Fig. 4:**
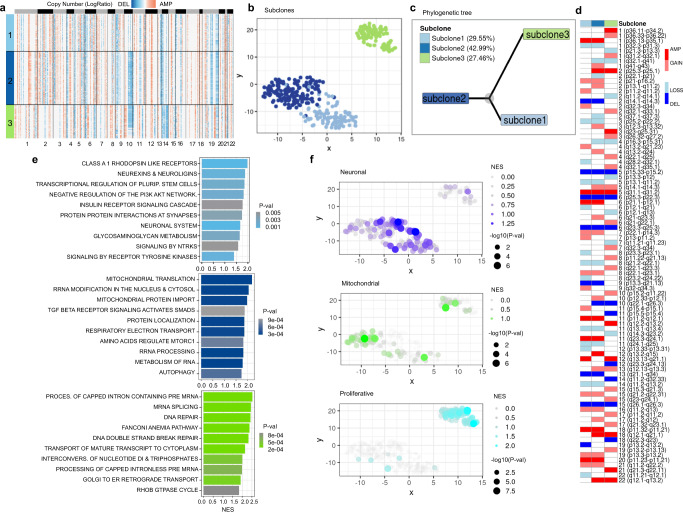
Deconvolution of the clonal substructure. **a** Clonal structure of sample BT1160 inferred by SCEVAN. **b** t-SNE plot of CNA matrix. **c** Inferred phylogenetic tree. **d** OncoPrint-like plot of BT1160 highlighting clone-specific alterations, shared alterations between, and clonal alterations. **e** GSEA was performed on REACTOME^36^ pathways for each subclone with a minimum size of 15 genes and a maximum size of 500 genes and with 10,000 as the number of permutations using the fgseaMultilevel function in the R package fgsea (v. 1.16), which calculates *P* values based on an adaptive multilevel splitting Monte Carlo scheme. **f** NES and −*log*_10_(*P* value) per cell of GBM cellular states^5^ computed by the Mann–Whitney–Wilcoxon single sample gene set test gene set implemented in the yaGST package^38^. Source data are provided as a Source Data file.

In sample BT1160, SCEVAN uncovers the presence of three tumor cell sub-populations, as shown in Fig. 4a, b. Phylogenetic reconstruction of the clone tree shows two close clones (subclones 1 and 2) and a significantly far third subclone (Fig. 4c). To better understand how individual clones fuel tumor growth and clonal selection, we investigated the reported alterations. SCEVAN identifies several truncal alterations, such as the amplification on Chr 5 (q23.2–q31.3), shared alterations, such as the deletion on Chr 10 (q22.1–q26.3), and subclone-specific alterations, such as the amplification in the green subpopulation on Chr 1 (q31.2–q32.1) and Chr 19 (q13.32–q13.33) (Fig. 4d). Interestingly, subclone-specific functional analysis reveals a differential activation of pathways that resemble a recent metabolic classification of Glioblastoma^5^. Subclone 1 (light blue) enriches pathways characteristic of the Neuronal subtype, subclone 2 (blue) has cells belonging to the Mitochondrial, and subclone 3 (green) contains cells with Proliferative/Progenitor subtype (Fig. 4e). Indeed, this finding is confirmed by the enrichment of individual cells for every subtype (Fig. 4f). The Proliferative/Progenitor subclone has several specific amplifications (1q21.3–q22, 1q31.2–1q32.1, 3q26.32–3q27.2, 4q32.1–4q35.1, 6p22.1, 8p11.22–8q21, 19q13.32–19q13.22). To identify drivers of the different cellular states, we performed differential analysis between genes with genomic coordinates in regions of the subclone-specific alterations. The top differentially expressed gene lying in the alterations specific to the subclone 3 was the Ubiquitin-conjugating enzyme E2T (*UBE2T*) gene, which is significantly up-regulated (*P* value 2.69*e*−43 log fold change 1.10) (Supplementary Fig. 10) enriching the activity of the pathway of DNA Repair. This gene encodes for the exclusive ubiquitin-conjugating enzyme (E2) that partners with the Fanconi Anemia (FA) ubiquitin ligase (E3). The E2T-FA complex is required for DNA interstrand crosslink repair as the monoubiquitination event implemented by E2T is essential for the recruitment of downstream DNA repair factors by FA^28^.

**Fig. 5:**
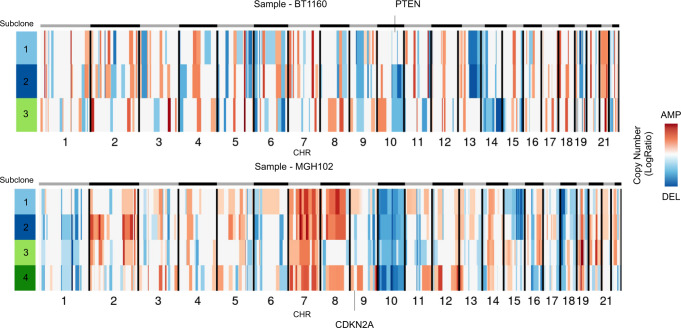
Tumor suppressor genes in the clonal substructure. Compact representation of clonal structure inferred with SCEVAN of scRNA-seq samples BT1160 and MGH102^7^, in which the alterations containing tumor suppressor genes *PTEN* and *CDKN2A* are subclonal. Source data are provided as a Source Data file.

Furthermore, the analysis of copy number substructure can characterize the clonal status of specific tumor-associated genes. SCEVAN reveals that in samples BT1160 and MGH102, alterations of tumor suppressor genes *CDKN2A* and *PTEN* are subclonal (Fig. 5). Indeed, in sample BT1160, the deletion on Chr 10 (q22.1–q26.3), containing *PTEN* (10q23.31), is shared between two out of three subclones, while in the remaining sub-population, this alteration is not present. Also, in the sample MGH102, the region 9p21.3 containing the gene *CDKN2A* is deleted in two of the four subclones. These results suggest that SCEVAN can resolve clonal copy number substructure in tumors from scRNA-seq data and identify subclonal differences and glioma-specific cancer states.

### Clonal evolution in multiregional GBM tumor

Glioblastoma heterogeneity has also been investigated in the spatial and temporal axes^22,29^ because a single biopsy may not be informative of the whole tumor. Multiple biopsies allow us to characterize the clonal architecture and evolutionary dynamics of GBM^30^.

**Fig. 6:**
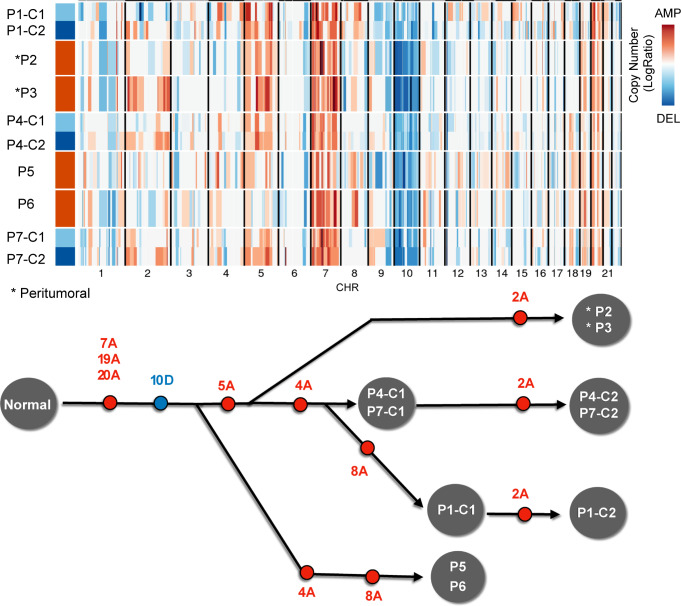
Temporal deconvolution of the clonal substructure. Compact representation of clonal structure inferred with SCEVAN of multiregional scRNA-seq samples of patient GS1^22^ and a phylogenetic tree deduced from clonal structure of the samples. Source data are provided as a Source Data file.

We used SCEVAN for the evolutionary analysis of clonal structure for multiregional scRNA-seq samples of GBM^22^. For example, we considered one case, GS1, with seven biopsies, two taken at the tumor periphery and the remaining at the core of the tumor. The clonal analysis of each sample with SCEVAN allows to infer an evolutionary tree of the clones (Fig. 6). Copy number alterations develop along several branches, and the peritumoral samples (P2/P3) are in a branch separated from the core samples, in which there is no amplification in chromosomes 4 and 8. Moreover, the amplification present on Chr 2 is clonal in peripheral samples and subclonal in some core samples (P1/P4/P7).

### Clonal structure of primary and metastatic lymph

**Fig. 7:**
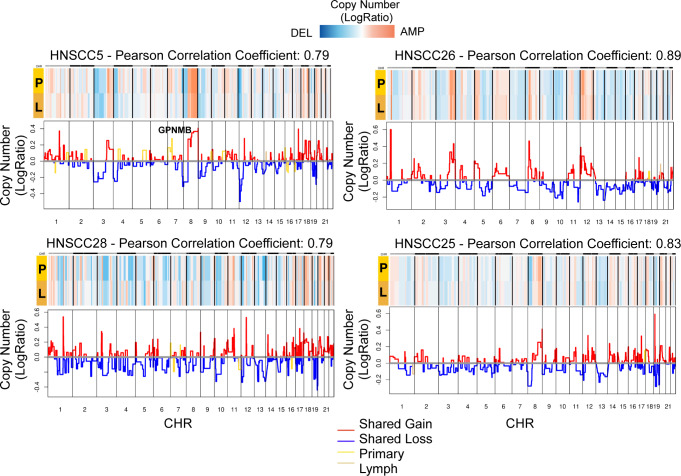
Clonal copy number comparison of matched primary and metastatic tumor. Copy number profile of primary (P) and metastatic lymph nodes (L) from samples of Head and Neck cancer dataset (HNSCC5, HNSCC25,HNSCC26, HNSCC28)^21^. Source data are provided as a Source Data file.

SCEVAN (and similar approaches) can address important questions, such as identifying similarities and differences between primary tumors and metastases. For this purpose, we considered primary HNSCC tumors and corresponding lymph node metastases^21^. Of the four considered cases, just one specific sample, the patient (HNSCC5), presented a different clonal structure between primary tumor and lymph node metastasis, particularly, the absence of amplification of chromosome 7 (p22.3–p13) in the lymph node metastasis, as shown in Fig. 7. Interestingly, this is the locus of Glycoprotein non-metastatic b (*GPNMB*) which is downregulated in lymph node metastasis (Supplementary Fig. 9). Furthermore, *GPNMB* increases tumor growth and metastasis in multiple contexts^31^. For the remaining patients (HNSCC20, HNSCC25, HNSCC26, HNSCC28) the clonal structure of the lymph node metastasis appeared to be the same as in the primary tumor. Therefore, we obtained a high correlation (Pearson correlation between 0.79 and 0.89) comparing the clonal profiles of the primary tumor and lymph node metastasis pairs. These data show that SCEVAN can be used to study the clonal evolution of metastatic cancer.

## Discussion

We described a variational segmentation approach to identify genomic copy number profiles from scRNA-seq data. The adopted joint segmentation algorithm is based on the notion that the cells in a given copy number clone share the same breakpoints. Thus, the expression profile of every individual cell, seen as a function of the genomic coordinates, contributes to the evidence of copy number alteration in each subclone. SCEVAN uses a set of stromal and immune signatures and the fact that malignant cells often harbor aneuploid copy number events to discriminate between transformed cells and microenvironment cells automatically. We used an extensive collection of annotated datasets of different tumor types confirming that SCEVAN is more accurate and faster than state-of-the-art methods. Our evaluation has shown that this approach is viable in cases with high purity and subjects with a significant amount of immune infiltration. Therefore, SCEVAN is particularly suited in studies where unsorted populations of single cells need to be analyzed to characterize, for example, the interaction between malignant cells and their microenvironment^6^.

The primary use of SCEVAN consists of delineating the clonal substructure in solid tumors based on differences in CNAs and studying the temporal and geographic evolution of tumors. In addition, we used SCEVAN to deconvolve the clonal structure of glioma tumors. For example, in one patient, we found the presence of cell populations with differential activation of glioma cellular states, confirming that the clonal architectures drive the heterogeneity of glioma subtypes^5^. Functional analysis of subclones revealed drivers of cellular states, such as the Proliferative/Progenitor (PPR) glioma subtype. We identified UBE2T as the top amplified and differential expressed gene in the PPR clone. Interestingly, UBE2T can be pharmacologically inhibited^32^, and therefore it results as a potential therapeutic target for PPR cells. Moreover, we have shown that with SCEVAN, we can characterize the clonal status of onco-suppressor genes such as *PTEN* and *CDKN2A*. Such characterization may be of interest for diagnostics or therapeutic targeting and for the exploitation of approaches based on synthetic lethality^33^. Clonal deconvolution extracted from scRNA-seq can also be used to study regional and temporal tumor evolution, as we have shown in the case of a multiregional GBM dataset, and for the characterization of the difference between primary and metastases.

SCEVAN has been evaluated here with different single-cell technology and recently used in a large study integrating millions of single cells from 538 samples and 309 patients across 29 datasets using the most commonly applied platforms such as 10x Chromium, Smart-seq2, GEXSCOPE, inDrop and Drop-Seq^34^.

Some limitations of our SCEVAN rely mainly on its basic assumption that their aneuploidy can identify cancer cells. However, there are cases such as liquid cancers (e.g., leukemia), pediatric cancers, Ependymomas, and others are known to harbor a minimal number of genomic alterations. Thus, our approach (and similar) may not be suited in this case.

## Methods

### Preprocessing of scRNA-seq data

The preprocessing phase is aimed at filtering out low-quality and irrelevant cells. Specifically, the cells with less than 200 detected genes and the genes expressed in less than 1% of cells are removed. The remaining genes are annotated by adding their genomic locations to the matrix using an Ensembl-based annotation package^35^ and then genes are sorted according to genomic coordinates. After annotation, the genes involved in the cell cycle pathway, obtained from REACTOME^36^, are filtered to reduce artificial segments caused by the cell cycle^11^.

### Identification of highly confident non-malignant cells

The input data *D* is an *m* × *n* single-cell gene expression matrix where *m* is the number of cells and *n* is the number of genes ordered by genomic positions. To segregate malignant from non-malignant cells, SCEVAN follows a multi-step approach. A small set of high-confidence normal cells is used to build a relative expression matrix and as a seed for identifying the cluster of normal cells. Then, the relative expression matrix is segmented and clustered as described in the following paragraphs. A set of gene signatures from public collections^6,37^, including cells of the tumor microenvironment, stromal and immune cells, such as lymphocytes, macrophages, microglial cells, dendritic cells, neurons, and others (Supplementary Data 1), is used to identify the high-confidence normal cells. We apply the Mann–Whitney–Wilcoxon single sample gene set test gene set implemented in the yaGST package^38^ and assume as normal confident cells the top classified cells with *P* value less than 10^−10^ and Normalized Enrichment Score (NES) greater than 1.0. We restrict the search to a maximum of 30 high-confidence non-malignant cells.

Then the copy number baseline, estimated from the median expression of confident normal cells, is removed from the count matrix, thus obtaining the relative matrix ${D}_{r}=D-{\tilde{{{{{{{{\bf{b}}}}}}}}}}^{T}$ where $\tilde{{{{{{{{\bf{b}}}}}}}}}$ is the *n*-dimensional vector with the median value of confident normal cells. If no confident normal cells are found, we assume that the sample is pure and contains only malignant cells. In this case, a synthetic baseline is removed from the malignant cells. The synthetic baseline is obtained by subtracting from each gene a random value extracted from a gaussian distribution with zero mean and the same standard deviation of the considered gene. To take into account the heterogeneity of the sample and to avoid smoothing CNV subclones, this step is applied to clusters of the count matrix. The number of clusters is automatically chosen by using the Calinski–Harabasz criterion, we use hierarchical clustering.

From now on, the relative gene expression matrix will be considered the sampled version of a function *u* defined on the genome with values in ${{\mathbb{R}}}^{m}$. In the case of single-cell data, the sampling is based on the relative expression values of each gene, in previous works, we have used a similar formalism for aCGH arrays^16^ where the sampling points are the position of each SNP probes, or for Whole Exome data^39^ the sampling points are the genomic positions position of exons.

### Edge-preserving smoothing

Before the segmentation phase, one of the key steps of SCEVAN is to smooth the relative expression function. Since the segmentation step described below assumes a piecewise-constant model of the copy number signal, we preliminarily proceed to perform a nonlinear smoothing of the gene expression along with the genomic coordinates to regularize the gene expression signal, reduce the outliers and at the same time to preserve the discontinuities which are the breakpoints between the copy number segments. We apply a filter grounded in the Bayesian framework of edge-preserving regularization,^40^ which considers the minimization of the total variation (TV) functional

1 $$\int\phi (|\nabla u|)$$

where *u* is the *m*-dimensional relative gene expression signal, ∇ *u* is its gradient and *ϕ*( ⋅ ) is a discontinuity-adaptive prior^41^. In particular, here we use $\phi (x)=\log \cosh (x)$, which has been shown to produce a well-posed minimization problem overcoming the non-differentiability of the TV at the origin^42^. The iterative numerical scheme implemented in SCEVAN is just the one-dimensional adaptation of the stable finite difference scheme previously reported^42^.

### Single-cell joint segmentation algorithm

SCEVAN uses a multichannel segmentation procedure that inputs all the cells in a given clone to identify the boundaries of homogeneous copy number. The procedure is based on the *Mumford and Shah energy* originally developed to analyze images. In their original work^12^, the authors introduced the basic properties of variational models for computer vision aimed at defining the mathematical foundations for appropriate decomposition of the 2D domain Ω of a vector-valued function ${{{{{{{{\bf{u}}}}}}}}}_{{{{{{{{\bf{0}}}}}}}}}:{{\Omega }}\to {{\mathbb{R}}}^{m}$ into a set of disjoint connected components (${{\Omega }}={\cup }_{i=1}^{l}{{{\Omega }}}_{i},\ \ \ {{{\Omega }}}_{i}\cap {{{\Omega }}}_{j}={{\emptyset}},\ \ \ 1\,\le \,i,j\,\le \,l,i\ne j$). The set of points on the boundary between the Ω_*i*_ is denoted as Γ. This partition is modeled such that the signal varies smoothly within a component and discontinuously between the disjoint components. This problem is known as piecewise smooth approximation. Here we adopt a special case of the Mumford–Shah model, when the approximation **u** of the signal **u**_**0**_ is constrained to be a piecewise constant function. This is best suited for CNV segmentation. In this case, the optimal segmentation is obtained by minimizing the following:

2 $$E(u,{{\Gamma }})=\mathop{\sum}\limits_{i}{\int}_{{{{\Omega }}}_{i}}{({{{{{{{{\bf{u}}}}}}}}}_{{{{{{{{\bf{0}}}}}}}}}-{{{{{{{{\bf{u}}}}}}}}}_{{{{{{{{\bf{i}}}}}}}}})}^{2}{{{{{{{\rm{d}}}}}}}}x{{{{{{{\rm{d}}}}}}}}y+\lambda|{{\Gamma }}|$$

where Γ is the boundary between the connected components Ω_*i*_ and ∣ ⋅ ∣ indicates its length and **u**_**i**_ is the restriction of **u** to Ω_*i*_. It is easy to show that the minimum for this model can be obtained by posing **u**_**i**_ as the mean of **u**_**0**_ within each connected component Ω_*i*_. Hence, this functional represents a compromise between the accuracy of the approximation and the parsimony of the boundaries. It is essential to notice that the resulting segmentation depends on the scale parameter *λ*. Indeed, it determines the number of computed regions: when *λ* is small many boundaries are allowed, and the resulting segmentation will be fine. As *λ* increases, the segmentation will be coarser and coarser.

In our case of segmenting the genome in regions of homogeneous copy number, we define a segmentation Γ = {*b*_1_, ⋯  , *b*_*M*+1_} as a set of ordered positions (breakpoints) partitioning the genome into *M* connected regions *R* = {*R*_1_, ⋯  , *R*_*M*_}. Each region *R*_*i*_ will contain all genes whose genomic coordinates lie between breakpoints {*b*_*i*_, *b*_*i*+1_}. We are modeling a function defined on a one-dimensional domain in Eq. (2), ∣Γ∣ reduces to the number of regions *M*. According to the original algorithm proposed in ref. ^16^, to minimize this function, adjacent regions *R*_*i*_ and *R*_*i*+1_ are iteratively merged in a pyramidal manner to create larger segments, and the reduction of the energy can be shown as:

3 $$E(u,{{\Gamma }}\backslash \{{b}_{i}\})-E(u,{{\Gamma }})=\frac{|{R}_{i}||{R}_{i+1}|}{|{R}_{i} |+|{R}_{i+1}|}||{{{{{{{{\bf{u}}}}}}}}}_{{{{{{{{\bf{i}}}}}}}}}-{{{{{{{{\bf{u}}}}}}}}}_{{{{{{{{\bf{i+1}}}}}}}}}|{|}^{2}-\lambda$$

where ∣*R*_*i*_∣ is the length of the *i*-th region, and **u**_**i**_ is a *m*-dimensional vector with the mean value of gene between *b*_*i*_ and *b*_*i*+1_, ∣∣ ⋅ ∣∣ is the *L*_2_ norm and \ is the set difference. To minimize (2), we follow a greedy procedure. We start with a segmentation having *n* regions, one for each gene. Then, at each step, we merge the adjacent regions that yield the maximum decrease of the energy functional upon merging. Since *λ* decides the end of merging, choosing an appropriate value is crucial to ensure the quality of the final segmentation. As in ref. ^16^, the selection for *λ* at each merging step is done dynamically, depending on two factors: the region’s size and the mean values of the consecutive regions being considered for the merge. Hence, the cost of merging two regions *R*_*i*_ and *R*_*i*+1_, associated with a breakpoint *b*_*i*_, is computed as follows:

4 $${\tilde{\lambda }}_{i}=\frac{\left|{R}_{i}\right|\left|{R}_{i+1}\right|}{\left|{R}_{i}\right |+\left|{R}_{i+1}\right|}||{{{{{{{{\bf{u}}}}}}}}}_{{{{{{{{\bf{i}}}}}}}}}-{{{{{{{{\bf{u}}}}}}}}}_{{{{{{{{\bf{i+1}}}}}}}}}{\parallel }^{2},$$

if ${\tilde{\lambda }}_{i} < \lambda$, the adjacent regions are merged and the *i*-th breakpoint removed. Otherwise, the energy function has reached a local minimum, and no merging can be done further. Therefore, *λ* is updated to the smallest of *λ*_*i*_ + *ϵ*, continuing the merging. The sequence of *λ* values is monotonically increasing as it corresponds to the amount of decrease of the energy functional at each step in (Eq. (3)). In ref. ^13^, we adopted a stopping criterion in such a way that the final segmentation is obtained when the increase in *λ* stabilizes and merging any further does not correspond to a significant decrease of the energy. The final stopping value is based on the variability of the adjacent region and the total variability of the data, *ν*. The total variability is computed as the sum of the standard deviation of all cells after the smoothing step. The stopping criterion is Δ*λ* = *λ*_*i*+1_ − *λ*_*i*_ ≤ *βν*, where *β* is a positive constant, representing the only parameter of the segmentation algorithm.

### Classification of malignant and non-malignant cells

The joint segmentation algorithm, applied to the relative gene expression matrix, returns a set of breakpoints and the interpolating function *u* minimizing (2), which is simply the mean gene expression between consecutive breakpoints in each cell. Hence, an intermediate CNA *m* × *n* matrix (*m* is the number of cells and *n* is the number of genes) is computed by substituting each expression value with the mean gene expression between consecutive breakpoints in each cell. This matrix is then clustered into two groups using hierarchical clustering. All the cells in the cluster containing the highest number of confident normal cells (if confident normal cells have been detected as described above) are then classified as non-malignant. The final CNA matrix is then obtained by subtracting the vector of the mean value of all the identified normal cells.

### Differential subclonal structure characterization

To deconvolve the clonal structure of a given sample, the CNA matrix containing just tumor cells is clustered using Louvain clustering^43^ applied to a shared nearest-neighbor graph^44^ (Fig. 1, step F). Each cluster represents a potential subclone. Therefore the joint segmentation algorithm is re-applied considering just the cells of the cluster (Fig. 1, step G). The segmentation results are classified with the CNV calling algorithm described below and analyzed to identify subclone-specific alterations, shared alterations between subsets of clones, and clonal alterations. Segments in each clone representing the same copy number alterations at genomic distances less 10*Mb* are first merged together. Afterward, two alterations in different clones are considered the same if the respective start or end breakpoints are at a genomic distance of less than 10 Mb and differ in size by less than 40%. The list of potential clone alterations is further filtered, retaining only clones having specific alterations.

### CNV calling

To obtain an estimate of the copy number status of each segmented region, we apply a mixture model-based algorithm to the mean expression level of each cell within each segment. This value is modeled as a mixture of five truncated normal distributions as in ref. ^45^. The parameters of the mixture are estimated using the EM algorithm^46^, starting from empirically chosen initial fixed parameters (Supplementary Table 1). Then each segmented region is classified in one of five copy number states deletion (0), loss (1), neutral (2), gain (3), or amplification (4). The final classification of each segmented region is obtained using the majority vote algorithm, starting from the classification for each cell in the relative segment.

### Comparison with other tools and analysis of bulk data

The raw count matrices of scRNA-seq samples reported in classification and copy number inference comparisons reported in the paper, are analyzed following the steps of SCEVAN Workflow (Methods) and with CopyKAT v1.0.5 and inferCNV v1.4.0. InferCNV was run using the author’s recommendations for the parameters denoise=TRUE, HMM=TRUE, HMM_type=’i6’, and cutoff=0.1 (for MM dataset)^17^, cutoff=1.0 (for multiregional GBM dataset)^4^.

The copy number variation profile from bulk biopsies was used as ground truth. In the case of Multiple Myeloma^17^, CNVkit v0.9.9 was used for segmentation. The integer Copy Number was assigned based on cutoffs specified in the CNVkit documentation (−1, −0.25, 0.2, and 0.7) (Supplementary Data 4). For the 26 Glioblastoma multiregional samples of low-depth whole-genome sequencing (WGS) on the bulk biopsies^22^, the copy number variations computed every 1-Mb window by Yu et al.^22^ was segmented using DNAcopy (v1.62.0)^47^ (Supplementary Data 5). The ground truth extracted from WES and WGS have of course different resolutions with respect to the single-cell data. Therefore, we first re-sampled the output of each method and the ground truth at the same genomic resolution. Specifically, for each position of the genome at 1-Mb distance we take the log ratio value or copy number integer value depending on the considered method. Then the Pearson correlation is computed between this re-sampled vector and the ground truth^11^. CNVkit and DNAcopy use circular binary segmentation (CBS), which is not used by any of the methods SCEVAN, CopyKAT, and inferCNV compared. This choice avoids a possible bias in the comparison.

For the comparison of breakpoints detection on synthetic data, we also use GenoCN v1.40.0 and the method doGFLars of jointseg v1.0.2. Since they do not have their own smoothing method, we use smooth.CNA of DNAcopy^47^ as previously suggested^24^.

The remaining parameters not mentioned are set as default parameters.

### Reporting summary

Further information on research design is available in the Nature Portfolio Reporting Summary linked to this article.

## Supplementary information

Supplementary Information

Description to Additional Supplementary Information

Supplementary Data 1

Supplementary Data 2

Supplementary Data 3

Supplementary Data 4

Supplementary Data 5

Reporting Summary

## Data Availability

The scRNA-seq data used in this paper are publicly available on the Gene Expression Omnibus (GEO): Colorectal cancer GSE132465^20^; Glioblastoma GSE131928^7^, GSE103224^10^, GSE117891^22^; Head and Neck Squamous Cell Carcinomas GSE103322^21^; Multiple Myeloma GSE223060^17^. All copy number variation profile from Bulk sequencing are available as Supplementary Information files, and raw data from multiregional GBM dataset^22^ of Bulk sequencing of genomic DNA is available at Genome Sequence Archive (GSA) under accession number HRA000179, upon request from the DAC. The synthetic data generated are made public at the following link https://zenodo.org/record/6628423. REACTOME pathway database is publicly available from Molecular Signature Database (MSigDB v7.4). Source data are provided with this paper.

## Source data

Source Data

## Code availability

SCEVAN is available in open source as an R package at the following address https://github.com/AntonioDeFalco/SCEVAN^48^.
