# Supplementary material for: A variational algorithm to detect the clonal copy number substructure of tumors from scRNA-seq data
Source: Nat Commun. 2023 Feb 25;14:1074. doi: 10.1038/s41467-023-36790-9 (PMC9968345; doi:10.1038/s41467-023-36790-9)
Supplement: Supplementary file 1 — Supplementary Information [file 41467_2023_36790_MOESM1_ESM.pdf]

	MIXTURE 0 (DELETION)	MIXTURE 1 (LOSS)	MIXTURE 2 (NEUTRAL)	MIXTURE 3 (GAIN)	MIXTURE 4 (AMPLIFICATION)
BOUND (Clonal Analysis)	$[\mu_c[1]^*3, \mu_c[1]-(3^*\sigma)]$	$[\mu_c[1]-(3^*\sigma), -\beta]$	$[-\beta, \beta]$	$[\beta, \mu_c[3]+(3^*\sigma)]$	$[\mu_c[3]+(3^*\sigma), \mu_c[3]*3]$
BOUND (Subclonal Analysis)	$[\mu_s[1]^*3, \mu_\mathrm{s}[1]-(3^*\sigma)]$	$[\mu_\mathrm{s}[1]-(3^*\sigma), -\beta^*2]$	$[-\beta^*2, \beta^*2]$	$[-\beta^*2, \mu_s[3]+(3^*\sigma)]$	$[\mu_s[3]+(3^*\sigma), \mu_s[3]*3]$
μ_c (Clonal Analysis)	$-\beta^*4$	$-\beta^*2$	0.0	β^*2	β^*4
μ_s (Subclonal Analysis)	$-\beta^*4$	$-\beta^*3$	0.0	β^*3	β^*4
σ	0.01	0.01	0.01	0.01	0.01
β	0.05	0.05	0.05	0.05	0.05
PRIOR	0.05	0.1	0.7	0.1	0.05

Supplementary Table 1. Initial parameters of the mixture of five truncated normal distributions for copy number classification.

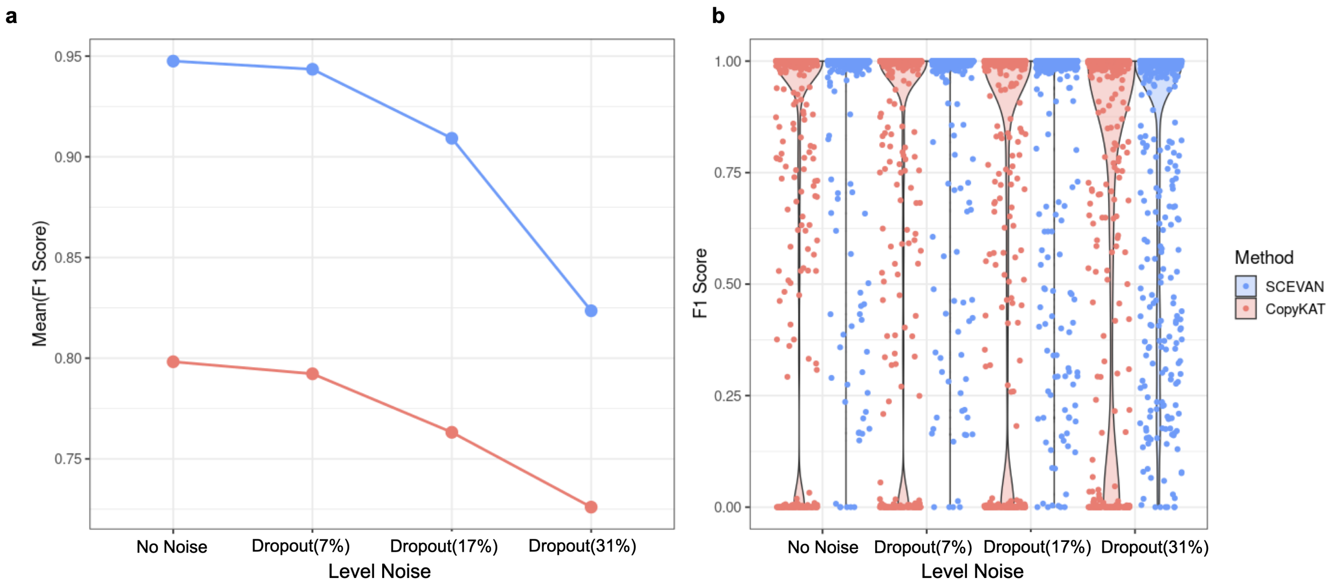

Supplementary Fig. 1. Benchmark of malignant cell classification task on synthetic data. a Comparison tumor/normal classification in terms of mean F1 score on 500 synthetic matrices, for various dropout noise levels, between SCEVAN and CopyKAT. (SCEVAN 0.948 0.943 0.909 0.824 - CopyKAT 0.798 0.792 0.763 0.726). **b** Violin Plots of the F1 score for 500 matrices at different noise levels using CopyKAT (Red) and SCEVAN (Blue).

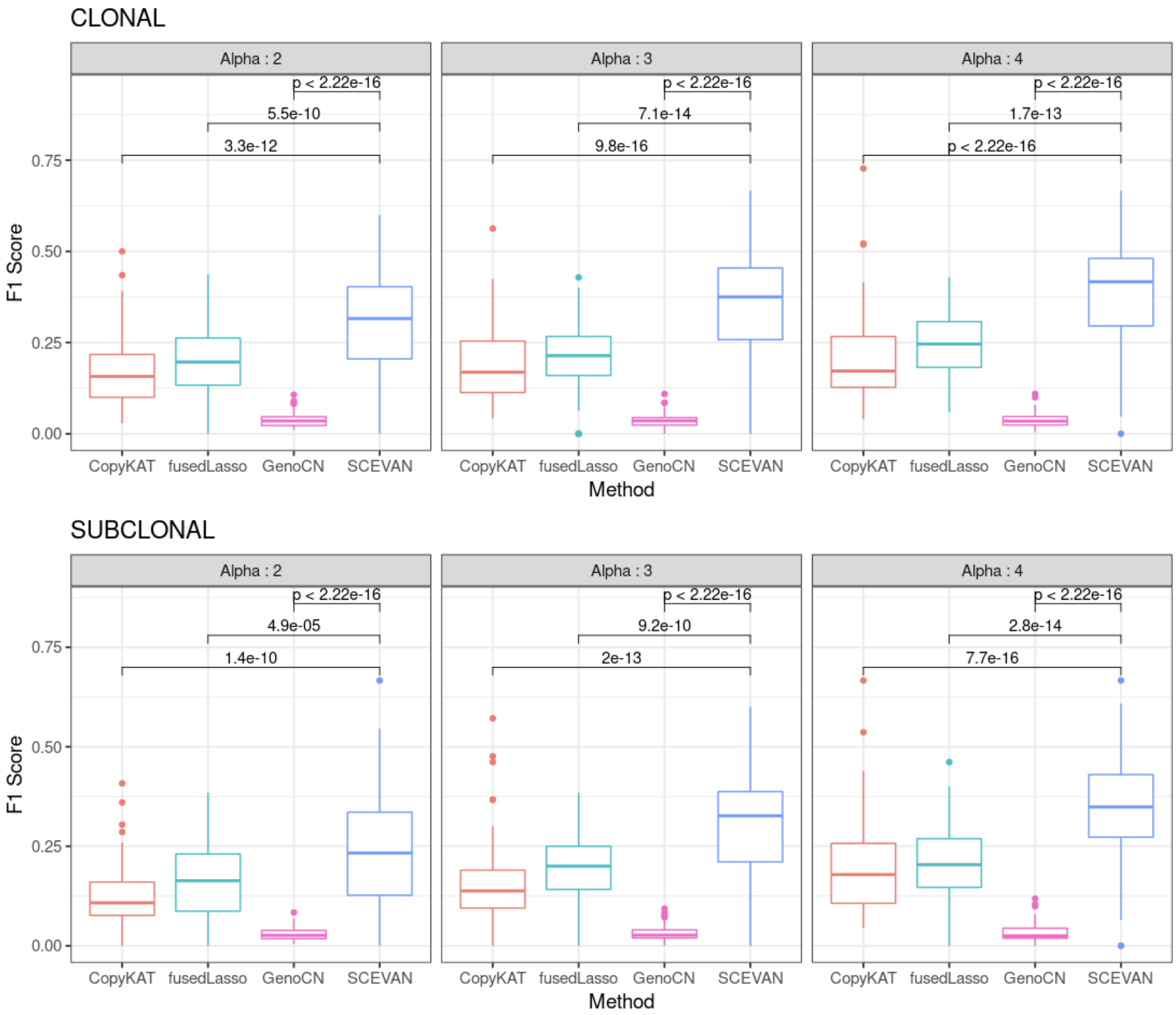

Supplementary Fig. 2. Benchmark of segmentation task on synthetic data. Comparison of segmentation accuracy in terms of F1 score with fixed tolerance threshold (20) as function of the magnitude of alteration α, and type of alteration, Clonal (Scenario I) and Sublconal (Scenario II). Box plots show the median as center, the lower and upper hinges that correspond to the 25th and the 75th percentile, and whiskers that extend to the smallest and largest value no more than 1,5*IQR. Values that stray more than 1.5*IQR upwards or downwards from the whiskers are considered potential outliers and represented with dots. Significance was computed by a one-sided Wilcoxon signed rank test.

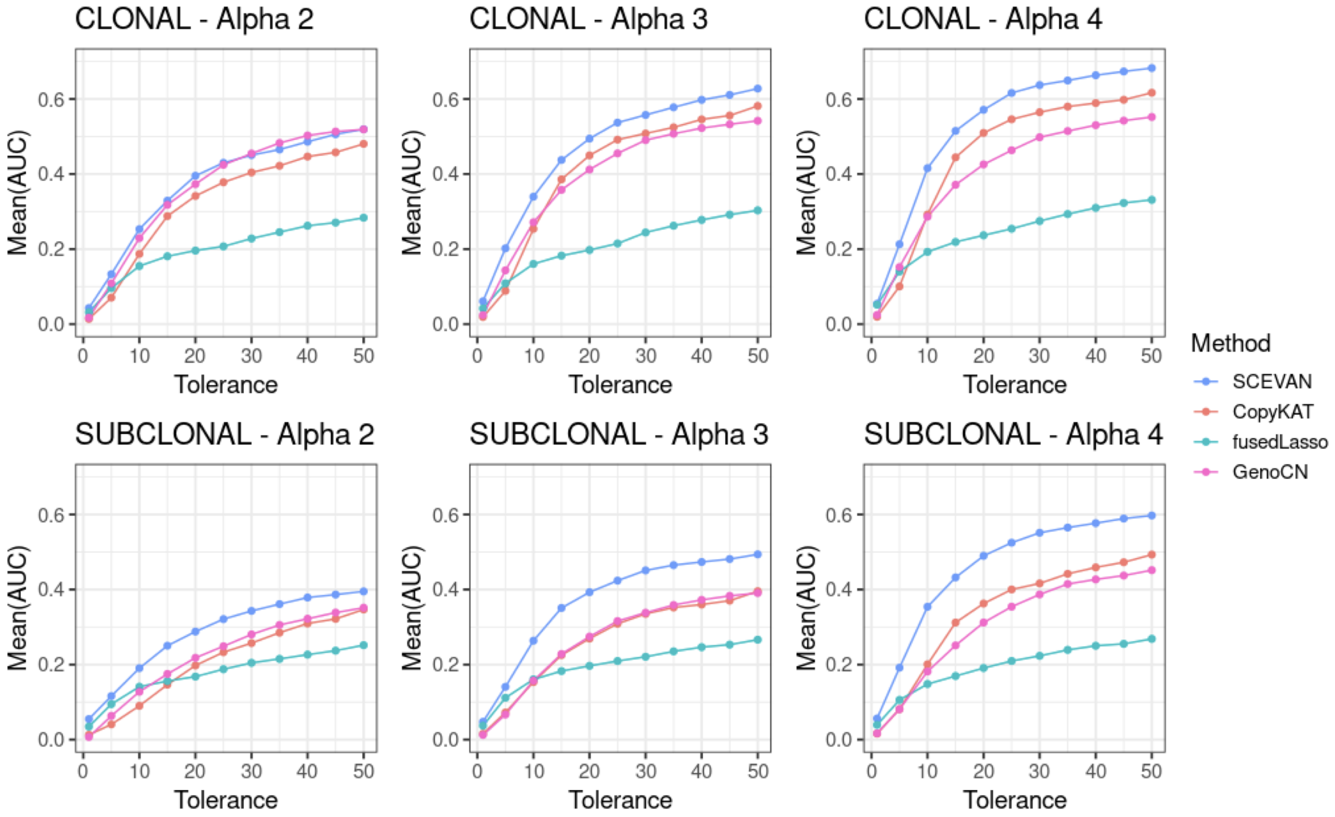

Supplementary Fig. 3. Segmentation accuracy on synthetic data. Comparison of segmentation accuracy in terms of mean PR AUC at different thresholds of tolerance on 100 synthetic matrices, for various magnitudes of alteration α, and type of alterations, Clonal (Scenario I) and Sublconal (Scenario II).

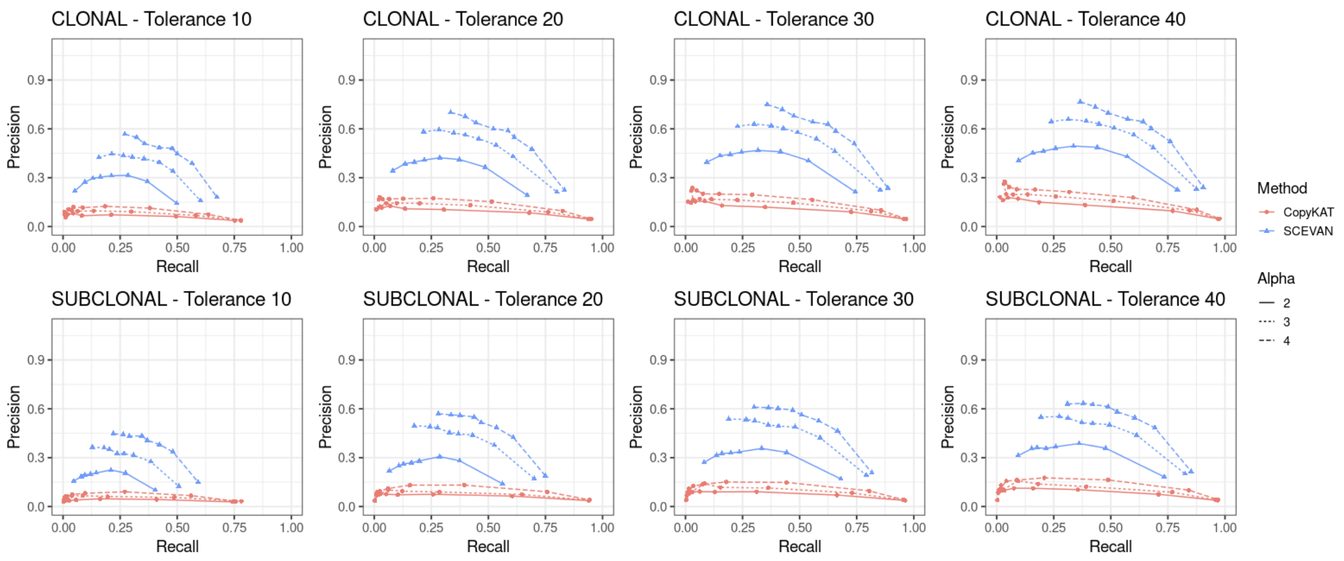

Supplementary Fig. 4. Precision-Recall of segmentation task on synthetic data at varying tolerance. PR curves obtained by varying the parameter β for SCEVAN and $KS.cut$ for CopyKAT in terms of magnitude of alteration α, level of tolerance (10,20,30,40) and type of alterations, Clonal (Scenario I) and Sublconal (Scenario II).

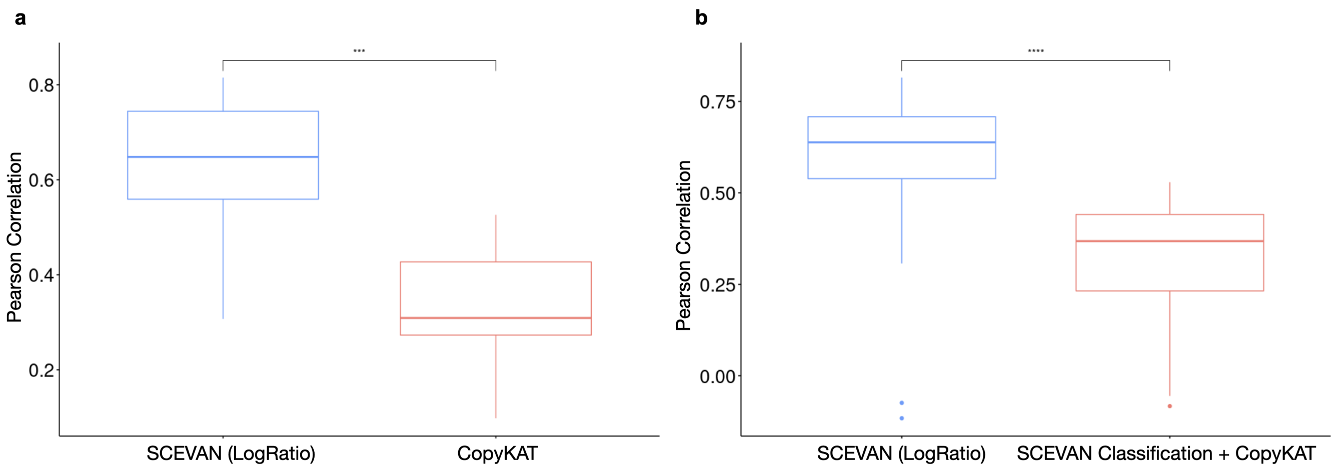

Supplementary Fig. 5. Comparison of the inferred copy number profile. In both **a** and **b** Box plots show the median as center, the lower and upper hinges that correspond to the 25th and the 75th percentile, and whiskers that extend to the smallest and largest value no more than 1,5*IQR. Values that stray more than 1.5*IQR upwards or downwards from the whiskers are considered potential outliers and represented with dots. Significance was computed by atwo-sided Wilcoxon signed rank test (***: p-value <= 0.001, ****: p-value <= 0.0001). **a** A correlation comparison between the inferred copy number of SCEVAN and CopyKAT with ground truth from low pass WGS data. The evaluation is only performed on samples in which CopyKAT has a good classification of tumor cells with an F1-score higher than 0.50. The 13 samples analyzed are S13P4, S3P4, S11P5, S13P6, S5P4, S13P5, S12P5, S6P2, S5P1, S6P5, S9P3, S1P2 and S3P8 from GBM multiregional dataset[1]. SCEVAN obtains a significantly higher correlation than CopyKAT, p-value $2.4e^{-4}$. **b** A comparison between the inferred copy number profile of SCEVAN and CopyKAT using as control cells the normal cells obtained from the SCEVAN classification. The evaluation is performed on 26 samples of GBM multiregional dataset[1] with the ground truth from low pass WGS data. SCEVAN obtains a significantly higher correlation than CopyKAT (p-value $1.3e^{-5}$), with a mean correlation of 0.57 against 0.33 of CopyKAT using SCEVAN classification as a reference.

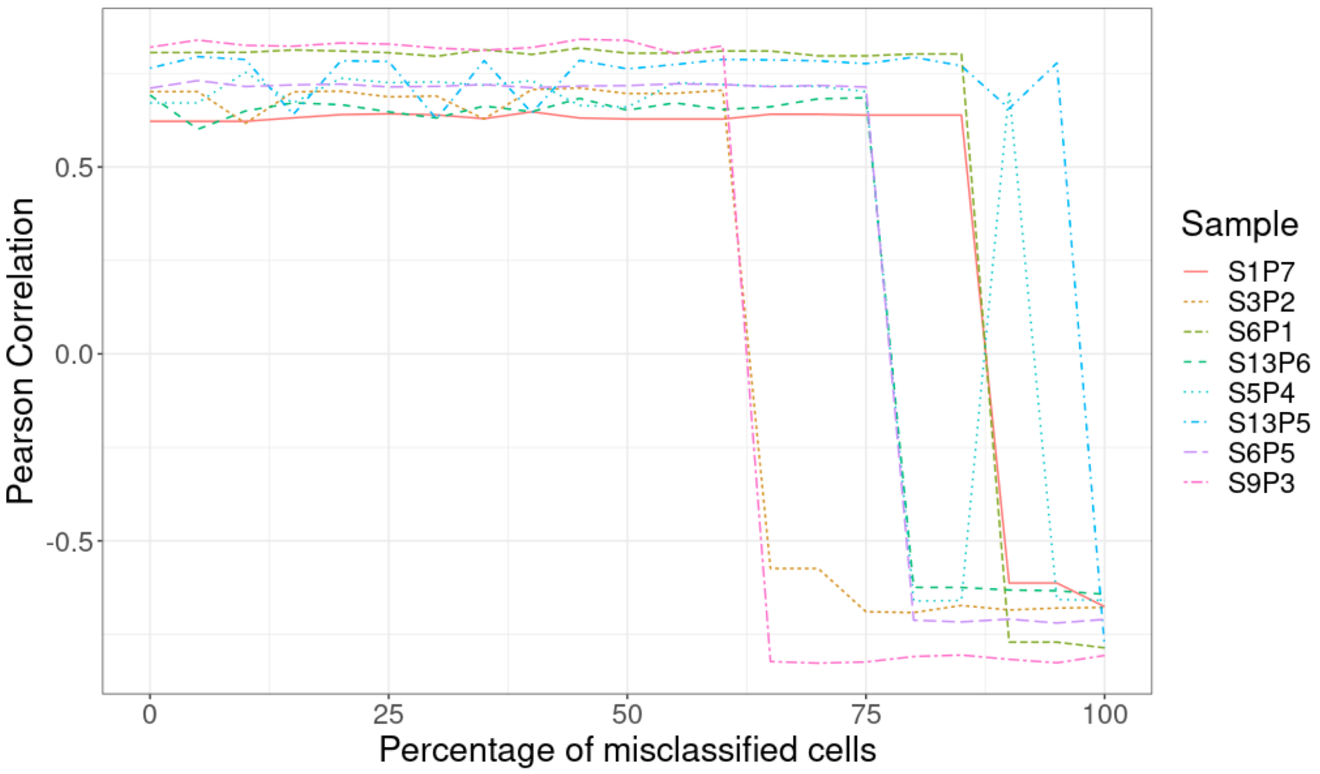

Supplementary Fig. 6. Correlation with ground truth varying misclassification errors. Correlation between the inferred copy number of SCEVAN with ground truth from low pass WGS data, to varying misclassification errors. The 8 samples are S1P7, SP32, S6P1, S13P6, S5P4, S13P5, S6P5 and S9P3 from GBM multiregional dataset[1].

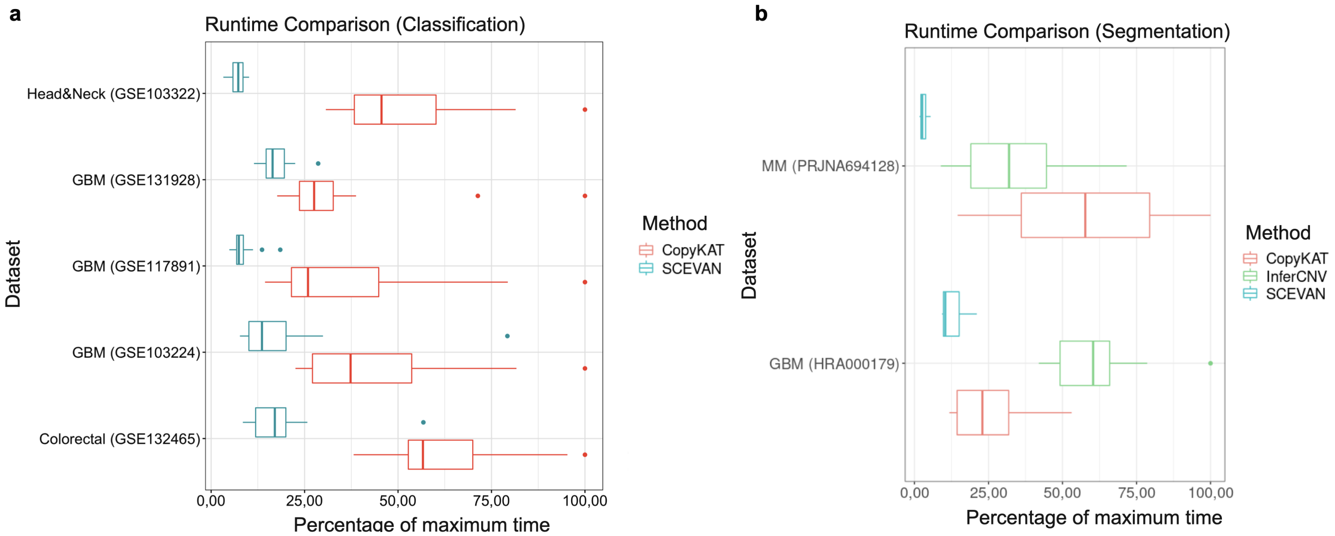

Supplementary Fig. 7. Runtime Benchmarking. In both **a** and **b** Box plots show the median as center, the lower and upper hinges that correspond to the 25th and the 75th percentile, and whiskers that extend to the smallest and largest value no more than 1,5*IQR. Values that stray more than 1.5*IQR upwards or downwards from the whiskers are considered potential outliers and represented with dots. **a** Classification time for each sample expressed as a percentage relative to the maximum time for each dataset Head and Neck Squamous Cell Carcinomas (GSE103322[2]) n = 5,717 cells examined over 20 scRNA-seq independent experiments, GBM (GSE131928[3]) n = 7,930 cells examined over 28 scRNA-seq independent experiments, GBM (GSE117891[1]) n = 2,957 cells examined over 25 scRNA-seq independent experiments, GBM(GSE103224[4]) n = 29,433 cells examined over 10 scRNA-seq independent experiments and Colorectal cancer (GSE132465[5]) n = 47,285 cells examined over 23 scRNA-seq independent experiments (Supplementary Data 2). **b** Runtime of copy number inference and segmentation for each dataset, expressed as a percentage relative to the maximum time, Multiple Myeloma (PRJNA694128[6]) n = 34,204 cells examined over 7 scRNA-seq independent experiments and Glioblastoma (HRA000179[1]) n = 2,957 cells examined over 25 scRNA-seq independent experiments.

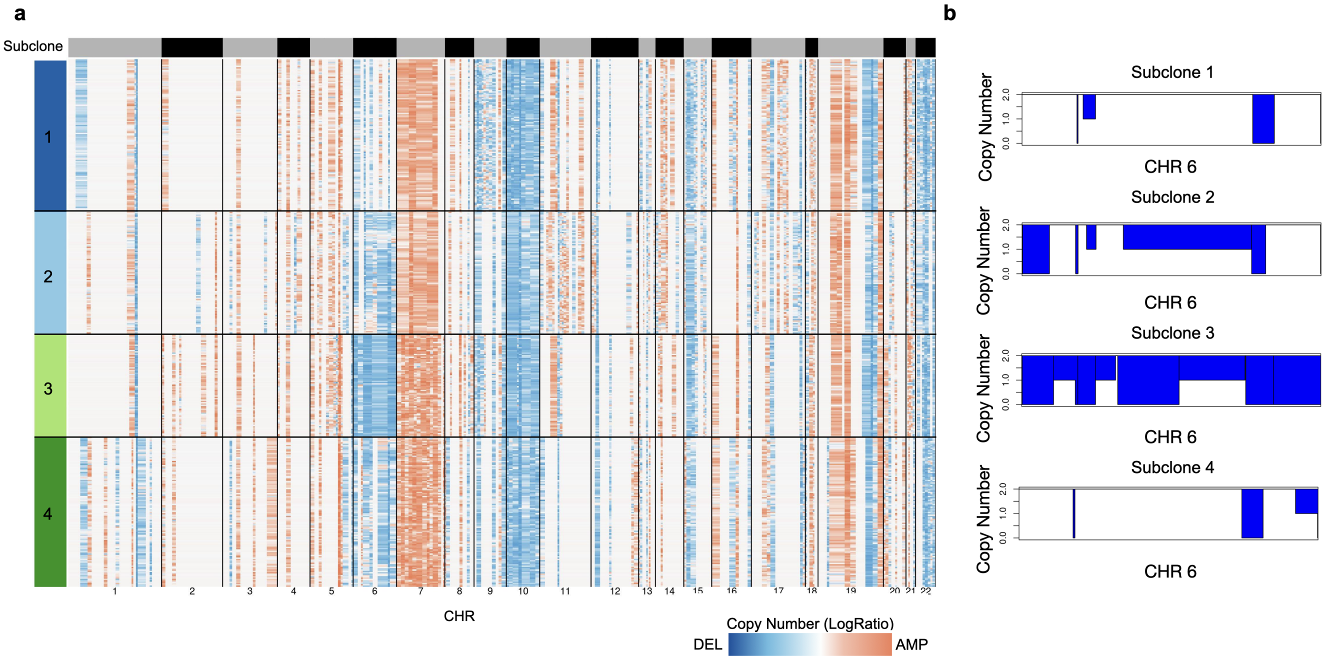

Supplementary Fig. 8. Subclonal copy number alterations of the chromosome. **a** Clonal structure of MGH105[3] inferred by clustering single-cell copy number profiles by SCEVAN. **b** Inferred copy number in chromosome 6 for each subclone.

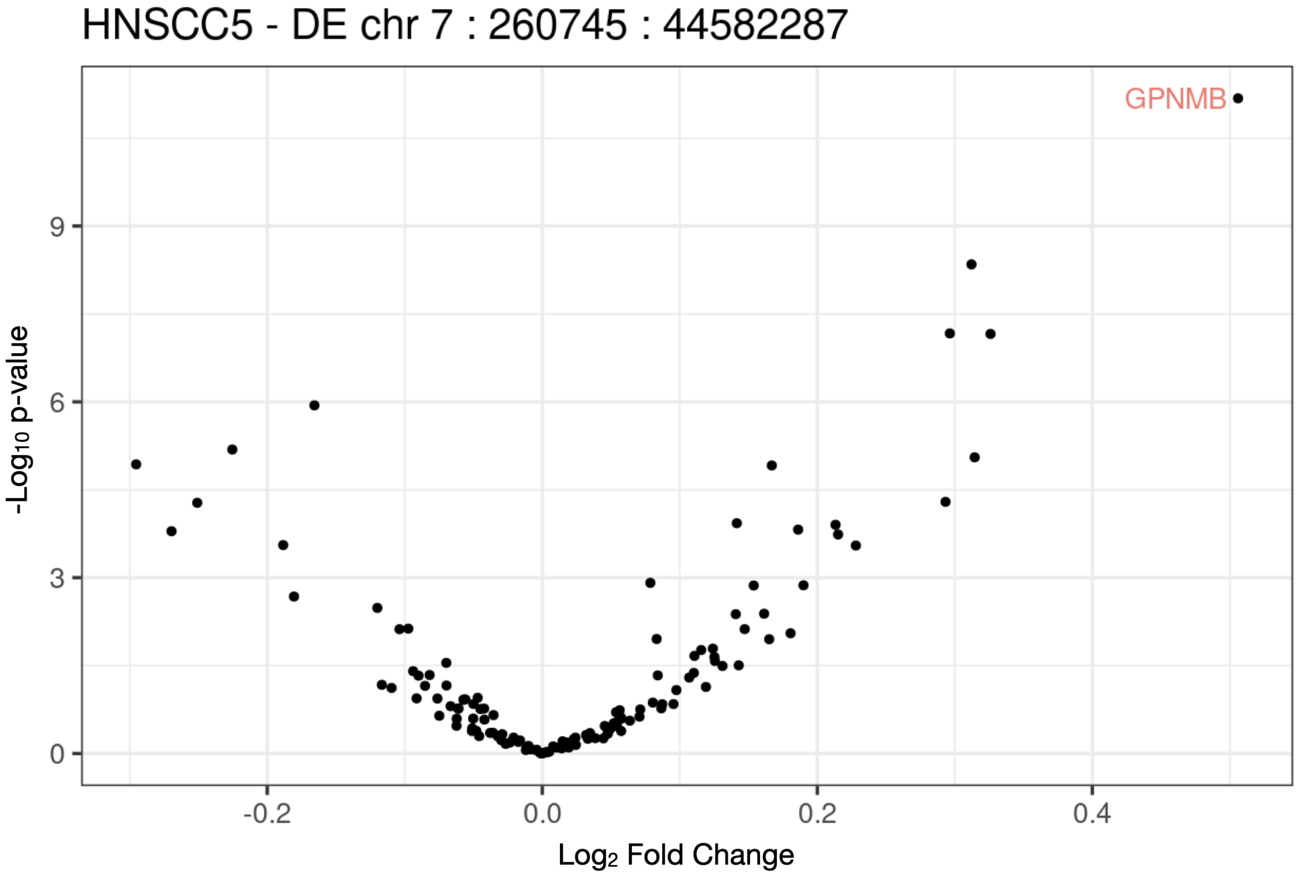

Supplementary Fig. 9. Differential analysis in specific copy number alteration. Differential analysis of genes belonging to the specific amplification on chromosome 7, between the gene expression of the primary tumor against the lymph node metastasis. Significance was computed by a two-sided t-test.

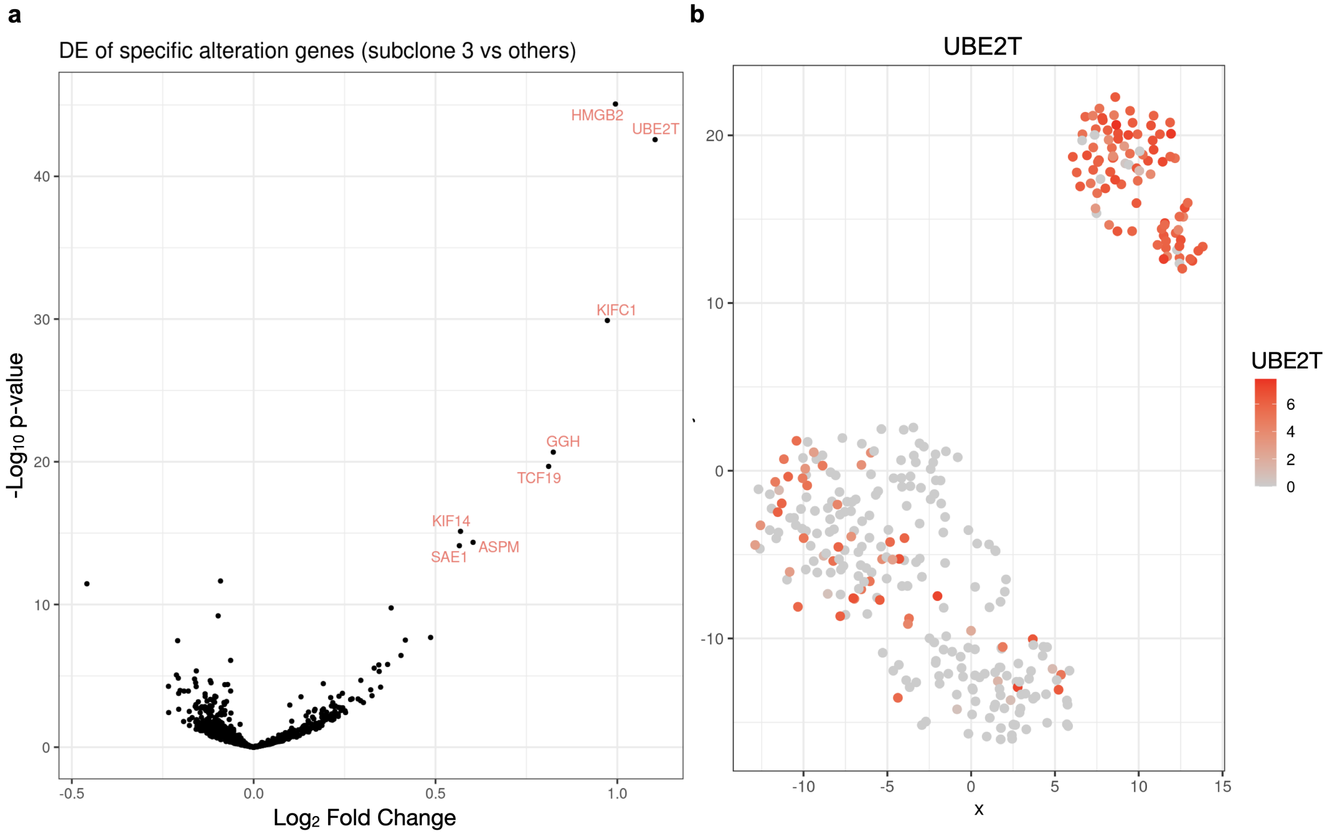

Supplementary Fig. 10. Top amplified and differential expressed gene of subclone. a Differential gene expression analysis of genes belonging to the specific amplifications of subclone 3, comparing subclone 3 against the others. Significance was computed by a two-sided t-test. **b** *UBE2T* expression on t-SNE plot of CNA matrix.

References

1. Yu, K. *et al.* Surveying brain tumor heterogeneity by single-cell RNA-sequencing of multi-sector biopsies. *Natl. Sci. Rev.* **7**, 1306–1318 (2020).
2. Puram, S. *et al.* Single-cell transcriptomic analysis of primary and metastatic tumor ecosystems in head and neck cancer. *Cell* **171** (2017).
3. Neftel, C. *et al.* An integrative model of cellular states, plasticity, and genetics for glioblastoma. *Cell* **178**, 835–849 (2019).
4. Yuan, J. *et al.* Single-cell transcriptome analysis of lineage diversity in high-grade glioma. *Genome medicine* **10**, 1–15 (2018).
5. Lee, H.-O. *et al.* Lineage-dependent gene expression programs influence the immune landscape of colorectal cancer. *Nat. Genet.* **52**, 594–603 (2020).
6. Liu, R. *et al.* Co-evolution of tumor and immune cells during progression of multiple myeloma. *Nat. Commun.* **12** (2021).
