# Supplementary material for: A variational algorithm to detect the clonal copy number substructure of tumors from scRNA-seq data
Source: Nat Commun. 2023 Feb 25;14:1074. doi: 10.1038/s41467-023-36790-9 (PMC9968345; doi:10.1038/s41467-023-36790-9)
Supplement: Supplementary file 2 — Description to Additional Supplementary Information [file 41467_2023_36790_MOESM2_ESM.pdf]

Description of Additional Supplementary Files

Supplementary Data 1: Signatures of normal cell types used to classify confident normal cells.

Supplementary Data 2: Description of scRNA-seq samples and classification results of malignant and non-malignant cells.

Supplementary Data 3: Description of synthetic scRNA-seq samples and classification results of malignant and nonmalignant cells.

Supplementary Data 4: Inferred copy number for 7 whole exome sequencing (WES) of Multiple Myeloma.

Supplementary Data 5: Inferred copy number for 26 low-depth whole-genome sequencing (WGS) of Glioblastoma multiregional samples.
