# Supplementary material for: A variational algorithm to detect the clonal copy number substructure of tumors from scRNA-seq data
Source: Nat Commun. 2023 Feb 25;14:1074. doi: 10.1038/s41467-023-36790-9 (PMC9968345; doi:10.1038/s41467-023-36790-9)
Supplement: Supplementary file 8 — Reporting Summary [file 41467_2023_36790_MOESM8_ESM.pdf]

nature portfolio

Corresponding author(s): Michele Ceccarelli

Last updated by author(s): Jan 18, 2023

Reporting Summary

Nature Portfolio wishes to improve the reproducibility of the work that we publish. This form provides structure for consistency and transparency in reporting. For further information on Nature Portfolio policies, see our Editorial Policies and the Editorial Policy Checklist.

Statistics

For all statistical analyses, confirm that the following items are present in the figure legend, table legend, main text, or Methods section.

n/a	Confirmed	
☐	☒	The exact sample size (*n*) for each experimental group/condition, given as a discrete number and unit of measurement
☐	☒	A statement on whether measurements were taken from distinct samples or whether the same sample was measured repeatedly
☐	☒	The statistical test(s) used AND whether they are one- or two-sided<br>*Only common tests should be described solely by name; describe more complex techniques in the Methods section.*
☒	☐	A description of all covariates tested
☐	☒	A description of any assumptions or corrections, such as tests of normality and adjustment for multiple comparisons
☐	☒	A full description of the statistical parameters including central tendency (e.g. means) or other basic estimates (e.g. regression coefficient) AND variation (e.g. standard deviation) or associated estimates of uncertainty (e.g. confidence intervals)
☐	☒	For null hypothesis testing, the test statistic (e.g. *F*, *t*, *r*) with confidence intervals, effect sizes, degrees of freedom and *P* value noted<br>*Give P values as exact values whenever suitable.*
☐	☒	For Bayesian analysis, information on the choice of priors and Markov chain Monte Carlo settings
☒	☐	For hierarchical and complex designs, identification of the appropriate level for tests and full reporting of outcomes
☐	☒	Estimates of effect sizes (e.g. Cohen's *d*, Pearson's *r*), indicating how they were calculated

Our web collection on statistics for biologists contains articles on many of the points above.

Software and code

Policy information about availability of computer code

Data collection	No software or code was used to collect data.
Data analysis	The custom code used to analyze data has all been deposited on GitHub and is available for public access: https://github.com/AntonioDeFalco/SCEVAN. The open source software used and described in Methods section are CopyKAT v1.0.5, inferCNV v1.4.0, CNVkit v0.9.9, DNAcopy v1.62.0, GenoCN v1.40.0, jointseg v1.0.2 and scDesign2 v0.1.0. This manuscript did not use commercial software or code.

For manuscripts utilizing custom algorithms or software that are central to the research but not yet described in published literature, software must be made available to editors and reviewers. We strongly encourage code deposition in a community repository (e.g. GitHub). See the Nature Portfolio guidelines for submitting code & software for further information.

Data

Policy information about availability of data

All manuscripts must include a data availability statement. This statement should provide the following information, where applicable:

- Accession codes, unique identifiers, or web links for publicly available datasets
- A description of any restrictions on data availability
- For clinical datasets or third party data, please ensure that the statement adheres to our policy

All single cell RNAseq data are available from Gene Expression Omnibus (GEO): GSE131928,GSE103322,GSE103224,GSE117891, GSE132465 and GSE223060. All copy number variation profile from Bulk sequencing are available as Supplementary Information files, and raw data from multiregional GBM dataset of

Bulk sequencing of genomic DNA is available at Genome Sequence Archive (GSA) under accession number HRA000179, upon request from the DAC. The synthetic data generated are made public at the following link https://zenodo.org/deposit/6628423 and there will be no restrictions on data availability. The REACTOME pathway database is publicly available from Molecular Signature Database (MSigDB v7.4) at the following link http://software.broadinstitute.org/gsea/msigdb.

Human research participants

Policy information about studies involving human research participants and Sex and Gender in Research.

Reporting on sex and gender	No data were collected in this study.
Population characteristics	No data were collected in this study.
Recruitment	No data were collected in this study.
Ethics oversight	No data were collected in this study.

Note that full information on the approval of the study protocol must also be provided in the manuscript.

Field-specific reporting

Please select the one below that is the best fit for your research. If you are not sure, read the appropriate sections before making your selection.

☒ Life sciences ☐ Behavioural & social sciences ☐ Ecological, evolutionary & environmental sciences

For a reference copy of the document with all sections, see nature.com/documents/nr-reporting-summary-flat.pdf

Life sciences study design

All studies must disclose on these points even when the disclosure is negative.

Sample size	No statistical methods were used to predetermine the sample size. The sample size was chosen according to the availability of data from public datasets.
Data exclusions	Low quality samples with no malignant cells were excluded from the public datasets used.
Replication	All attempts to replicate the analysis were successful.
Randomization	There are no experimental groups in this study.
Blinding	There are no experimental groups in this study.

Reporting for specific materials, systems and methods

We require information from authors about some types of materials, experimental systems and methods used in many studies. Here, indicate whether each material, system or method listed is relevant to your study. If you are not sure if a list item applies to your research, read the appropriate section before selecting a response.

Materials & experimental systems

n/a	Involved in the study	
☒	☐	Antibodies
☒	☐	Eukaryotic cell lines
☒	☐	Palaeontology and archaeology
☒	☐	Animals and other organisms
☒	☐	Clinical data
☒	☐	Dual use research of concern

Methods

n/a	Involved in the study	
☒	☐	ChIP-seq
☒	☐	Flow cytometry
☒	☐	MRI-based neuroimaging
